# A one-pot rotational DC-bipolar approach for fabricating artistic metallic carpets

**DOI:** 10.1038/s41598-022-20929-7

**Published:** 2022-10-03

**Authors:** Fereshte Gholami, Mojtaba Shamsipur, Afshin Pashabadi

**Affiliations:** Department of Chemistry, Razi University, Kermanshah, Iran

**Keywords:** Chemistry, Engineering, Materials science, Nanoscience and technology

## Abstract

This is a brief report on the fabrication of concentric multi-element metallic carpets through a one-pot rotational bipolar electro-engineering procedure. A suspended piece of nickel foam as a bipolar electrode (BPE) is rotated in an aqueous solution containing a ternary mixture of metal ions when sufficient DC potential is applied to driving electrodes. The customizable tools of this art are potential gradient, rotation, and concentration/kinetic polarizations. Creating the multi-element radial gradient is typically tested in a one-pot artistic jewelry electroplating.

## Introduction

One-pot electrodeposition (electroplating) is a facile approach for the deposition of dissolved electroactive ionic species, specifically metal ions, on conductive substrates for the fabrication of various types of two-/three-dimensional materials coatings^1–3^. One of the limitations of electroplating is the impossibility of creating gradients of materials (isolated material zones) perpendicular to the applied field, which arises from the uniformity of the applied potential across the working electrode in conventional electrochemistry.

Bipolar electrochemistry (BE) affords a potential gradient across the bipolar electrodes (BPE) immersed in electrolytes with no direct electrical connection. The applied interfacial potential difference drives redox reactions on extremities (poles) of BPE. The upgraded forms of this ability in the electrolyte containing electroactive metal ions with a stationary floating conductor will be useful in wireless electroplating^4–14^. Upon implementing a sufficient DC potential to driving electrodes, due to the creation of a potential drop across the driving electrodes, a linearly decreasing potential difference between the extremities of the suspended object is generated that drives opposite redox reactions at both sides of BPE^15–18^.

The major drawback in the already bipolar electroplating studies^19–31^ is the impossibility of the intermittent change of the bipolar cathode/anode position thus the electrodeposition only occurs at one pole of the BPE. To this end, a spatiotemporal polarity change of the anode/cathode poles^32–34^ can be attained through an AC power supply to implement an alternating potential to the driving electrodes. Over the last decade, some research works explored the control motion of wireless conducting objects to enable different types of self-propulsion^35–39^. A more exhaustive and customizable approach that permits applying the interfacial gradient potential to the entire 360° of BPE margin is the rotation of BPE using a motor controller and a DC power supply. It allows applying a uniform, successive and consistent potential gradient throughout BPE. The electroplated composite prepared by this method delivers a concentric multi-element composition of the isolated metal alloys^40^.

In this work, the adjustment of bipolar gradient potential as a function of applied DC potential, the rotation speed of BPE, inherent kinetic polarization of the metal ions (standard reduction potentials) and concentration polarization (concentration of precursors) generate a combinational gradient potential sensed by the rotated-BPE, endowing a concentric isolated alloy across the center-to-borders of BPEs. We exploited this methodology in an artistic one-pot electro-engineering of two typical ternary mixtures of Cu-Ni-Mn and Cu-Co-Mn at nickel foam (NF) as the bipolar electrode.

## Results and discussion

A two-dimensional carpet-resemble isolated concentric metallic zones of Cu-Ni-Mn are typically prepared through rotational DC-bipolar electroplating. A constant DC potential (from 4 to 12 V) was applied between a pair of stainless-steel driving electrodes with a separation distance of *ca.* 2.5 cm, as the length of the bipolar cell. One piece of NF (10 × 12 mm) as a typical BPE was connected to the shaft of a motor controller, immersed in the middle of the BP cell containing a particular solution of the metal ions, and was rotated at a constant speed of 100 rpm (further information on the experimental section is in SI). Figure S1 discloses the place of attachment of BPE to the rotator tip that remained unchanged (the upper side is designated in Fig. S1). Since the concentric gradients formed at both sides of BPE are symmetrical, this issue can be resolved by exchange of the attachment place from one side to the other side of BPE at half-time of electroplating. Considering the existence of a potential gradient from the edge to the center of BPE, selecting variable concentrations of the electroactive metal ions with different standard reduction potentials allows the control of kinetic and concentration polarizations to control the fabricated fade concentric gradient across the BPE. Moreover, the polarity changes through rotation allowing the one-pot electro-engineering of the metallic carpet. Before anything, we aimed to explore the actual role of anodic and cathodic poles of BPE on electroplating and electrodissolution of the metallic layers. To this end, a facile static bipolar electroplating was accomplished to distinguish the role of the anode and cathode of BPE in the formation of metallic carpets. The linear metallic gradient was just formed at the cathodic side, as shown in Fig. S2. The possible anodic dissolution of deposited layers at cathodic layers was also studied through static electroplating (at 8 V) of metal ions separately on three distinct nickel foam (Fig. S3). After cathodic electroplating, the position of bipolar poles was exchanged through 180° rotation of BPE to consider possible dissolution of the electroplated layers. For the Cu layer, after rotation, the primary deposited layer on edge of BPE dissolved at the anodic potential (see Fig. S3). In the case of Ni, the dissolution was lower, while for the Mn, the deposited cathodic layer did not change, confirming a gradual diminishing trend of Cu > Ni >  > Mn. This relative trend is determinant along the rotational electroplating of Cu, Ni and Mn.

**Figure 1:**
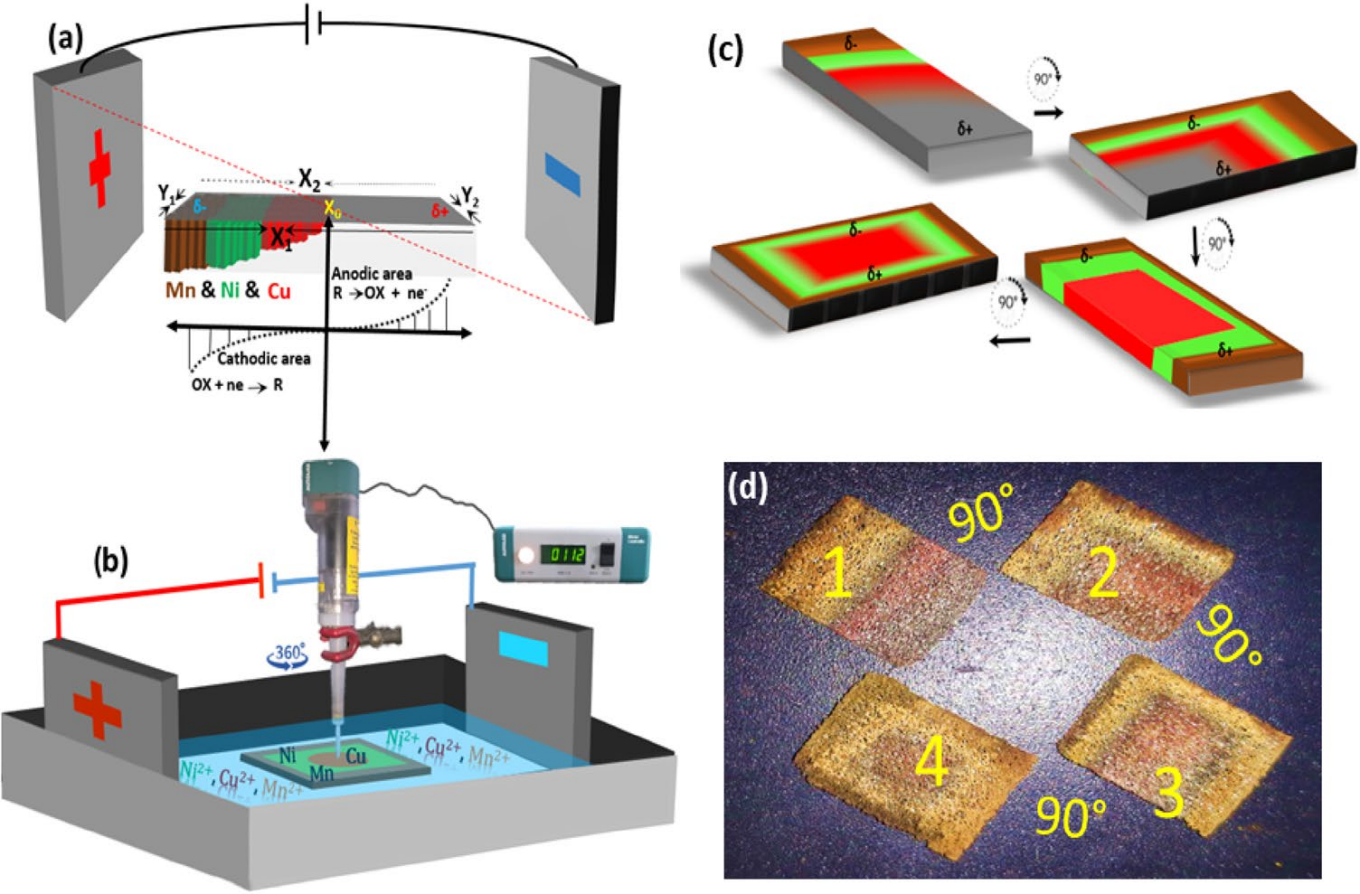
(**a**) Potential distribution within the BPE. (**b**) Schematic presentation of the set-up used for the bipolar electrodeposition of Cu–Ni–Mn gradients. The formed concentric pattern are identical on both sides of BPE, although, a centric place of rotor attachment remained unchanged at the upper face of nickel foam, see Fig. S1). (**c**) Schematic presentation of dependency of electrodeposition on the BPE orientation. (**d**) The photograph was taken from four manual rotations of the BPE (90° to 360°).

One of the main discriminators is the maximum potential sensed by the extremities of the BPE, in which the electroactive species with high reduction potentials (Mn) can be easily deposited just on the edges of the negatively charged BPE (during the cathodic half-cycle of the rotating BPE)^1^, as well as, the anodic dissolution of Cu and Ni that intensifies the isolation of the individual metallic zones. The overpotential along the BE gradually reduces by moving toward the center of BPE thus the cations with lower-reduction overpotentials (like Ni^2+^ and Cu^2+^) can deposit at the middle and center of the BPE, respectively (as shown in Fig. 1a). The rotation of the rectangular BPE around a central axis between the driving electrodes leads to AC-bipolar electrochemistry (Fig. 1b). The polarity change originated from the 360° rotation affords a uniform concentric gradient along all four sides of the BPE, enabling electroplating of the isolated metallic zones in an ellipse-like form. To illustrate an in-depth picture of the changes, the rotational speed was practically slowed down through a four manual rotation-section 90° of the bipolar electrode (Fig. 1c). The equivalent photographic image of this schematic is presented as Fig. 1d. The longitudinal faces (X_1_ and X_2_) and transverse faces (Y_1_ and Y_2_) are marked in Fig. 1a. By implementing the first reducing gradient potential on Y_1_, the first linear metal composing was attained. Upon rotation from 0 ͦ to 90 ͦ, the Y_1_ face is replaced by X_1_ and, thus, a cross-sectional ternary (e.g., Cu, Ni and Mn) electrodeposition can be formed. In the same way, the rotation of the different faces of BPE through rotation degrees of 180 ͦ, 270 ͦ and 360 ͦ make possible the formation of triple intersecting gradients. The photographic images inserted in Fig. 1c show a rectangle radiant of the metallic zone with sharp corners. Meanwhile, the minimum rotation rate of 100 rpm provides ellipse-like concentric metallic zones.

The potential-difference across the BPE (ΔE_BPE_) drops linearly along BPE as a fraction of E_tot._ It can be directly estimated from the Eq. (1)^17^:

1 $${\Delta E}_{BPE}={E}_{tot}\times \left[\frac{{L}_{BPE}}{{L}_{channel}}\right].$$

Here, E_tot_ is the externally applied potential between the two driving electrodes, L_BPE_ is the length of BPE (bare NF), and L_channel_ the distance between the driving electrodes (stainless steel plates).

**Figure 2:**
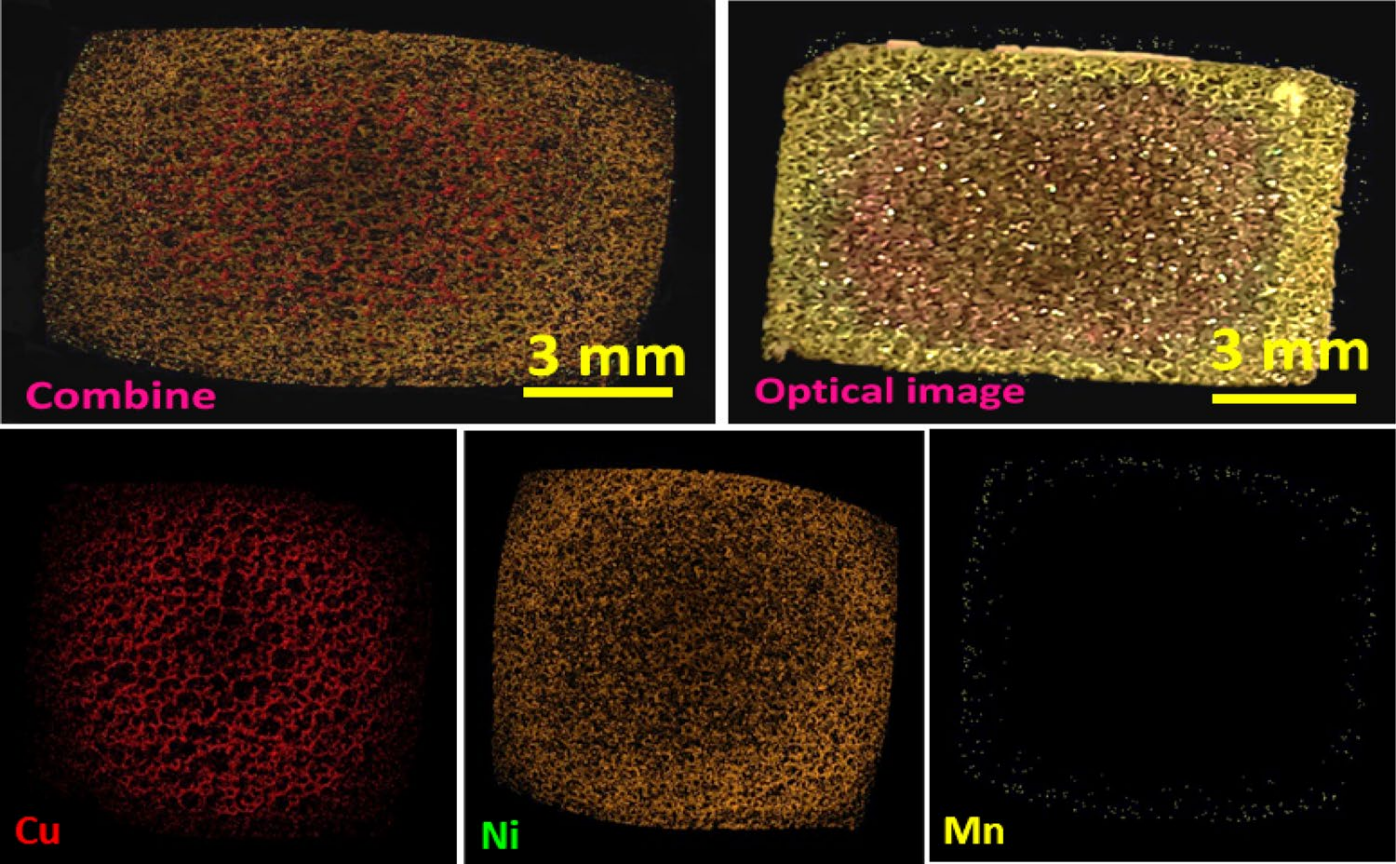
The optical and equivalent mapping analyses taken from metallic carpet formed by applying a potential of 4 V at rotating rate of 100 rpm, concentration of Cu, Ni and Mn nitrates in bipolar cell were 0.02,0.2 and 0.4 M, respectively. The corresponding EDX spectrum are in Fig. S4.

We first applied a non-optimized potential of 4 V as the minimum operating potential to the driving electrodes for a specific time. The optical images and corresponding elemental mappings based on energy-dispersive X-ray spectroscopy were taken from NF electrodes to disclose how to arrange the created metallic carpet array on the NF-BPE surface. The equivalent EDX spectrum is presented in Fig. S4. The results show improper isolation on the metallic zones created at 4 V (Fig. 2). At such a low overpotential of 4 V, Cu is the predominant form deposited throughout the central region to near the margins, and, Ni is more likely to deposit on the edges, while the visual inspection shows no Mn deposition, and low anodic dissolution of the electroplated Cu layer (a bimetallic carpet forms).

**Figure 3:**
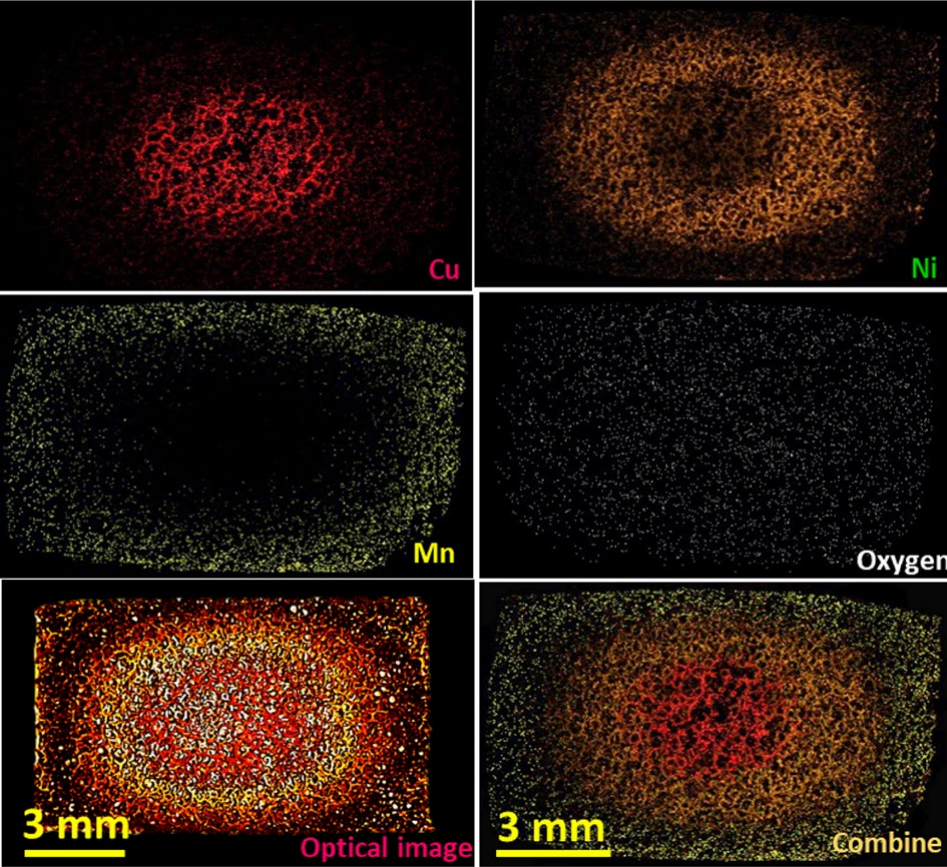
The optical and equivalent mapping analyses taken from the metallic carpet of Cu, Ni and Mn formed by applying a driving potential of 8 V. The corresponding EDX spectrum is shown in Fig. S5.

The ternary metallic carpets of Cu, Ni and Mn are obtained at higher applied DC-potentials, as big as 8 V. By increasing the potential from 4 V, the Mn deposition increases, and it is completely observable at 8 V (Fig. 3). The results of mapping analyses demonstrate a uniform pattern of oxygen throughout the BPE, indicating an appealing-colored combination of the metal-oxides. The formation of oxides was typically shows in mapping analysis and approved by EDX spectrum shown in Fig. S5. Due to the positive standard reduction potential, it is expected copper covers the entire NFBPE. Nevertheless, the manipulation of ion concentration and the applied potentials permit confining the effect of standard potentials to achieve a one-pot compositional gradient.

**Figure 4:**
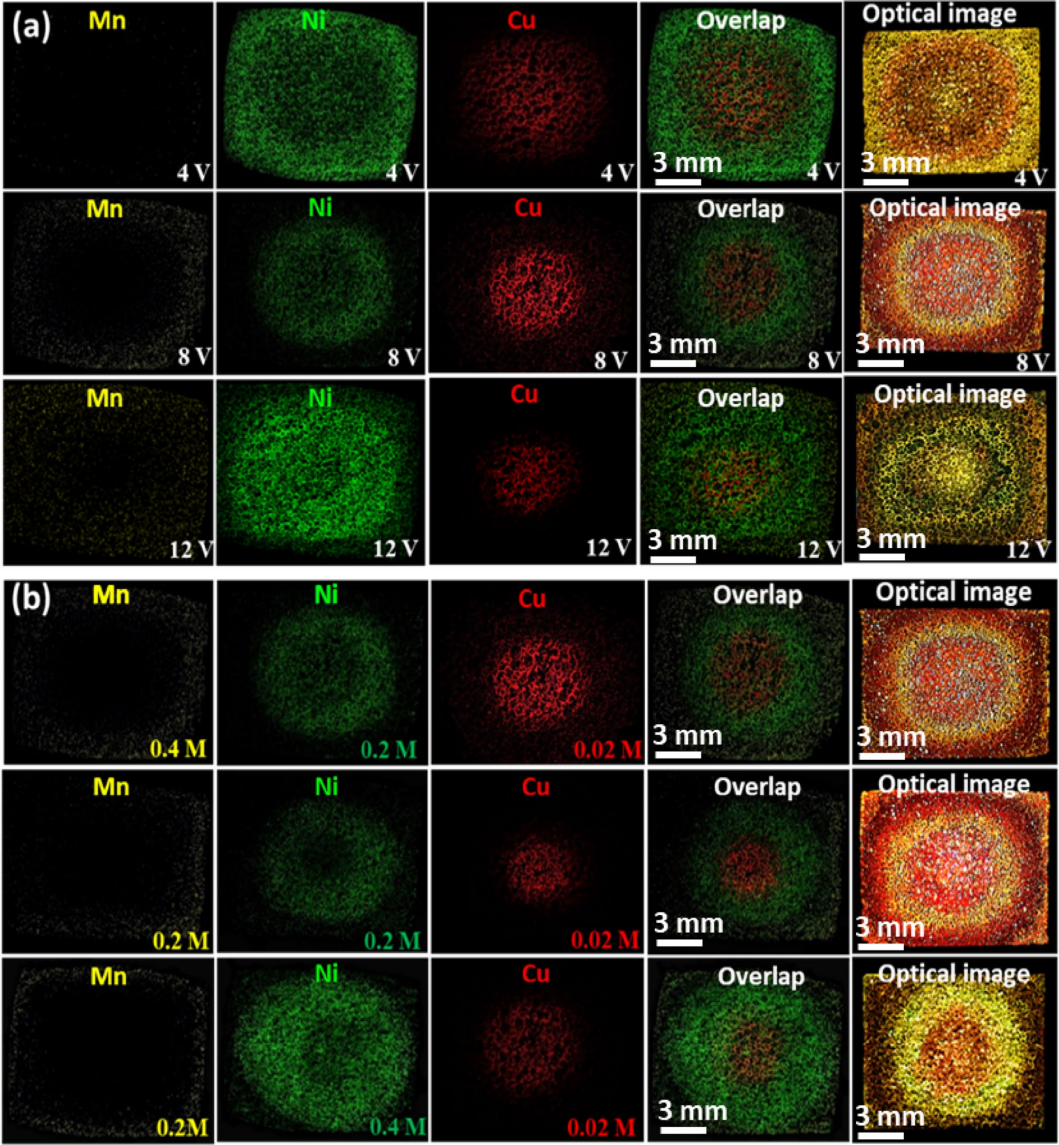
The optical and equivalent mapping analyses taken from metallic carpets formed at (**a**) different applied potentials and (**b**) different sets of metallic ions concentration.

The effect of the applied potential was studied on the fabrication of the metallic carpets at the different dc potentials of 4, 8 and 12 V. The optical images and the corresponding mapping analyses are compared in Fig. 4 to unravel the possible deposition at each zone, elemental patterning and the zone expansion (isolation). As shown in Fig. 4a, at 4 V, no observable manganese was deposited. While the edges of BPE are entirely occupied by Ni, confirming a sufficient potential for Ni reduction at the borders and insufficient for the Mn deposition. Further, upon the increase of potential to 8 V, Mn replaces Ni and deposits at the margin of BPE. At 12 V, the amount of the deposited Mn increases and expands toward the middle zone, such a behavior observes for the electroplated Ni, too. In all these cases, the Cu zone is restricted to the center of BPE with minimal changes, although, upon increasing potential, it gets a smaller diameter, which could be justified by the increase of the absolute value of the anodic potential toward the center of BPE (see Fig. 4a). Indeed, the rotation of NF will expose the Cu-zone to greater positive potentials and the siege ring of the positive potential at anodic half-cycle becomes tighter and tighter as the DC potential increases. The visual inspection of NF indicates the formation of exclusive metallic carpet engineered at each potential over the entire studied potential range of 4 to 12 V, however, the more isolated metallic carpets were obtained at 8 V compared to 4 and 12 V.

**Figure 5:**
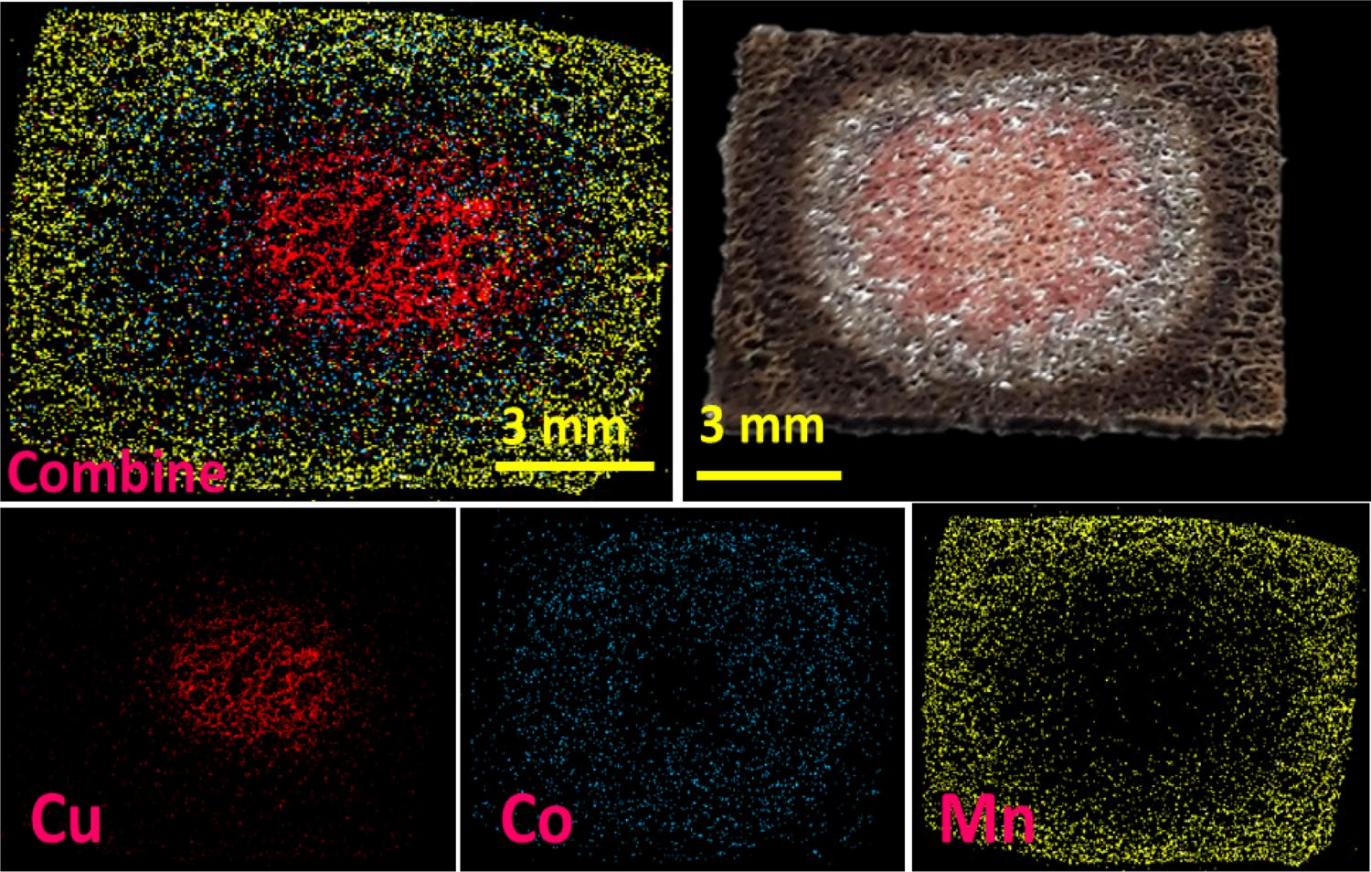
Mapping result of the Cu–Co–Mn metallic carpet formed by applying a potential of 8 V. The equivalent EDX spectra are presented in Fig. S7.

The effect of concentration polarization was investigated on the expansion of the isolated zones and thermodynamics of deposition, which allows the altering net potential sensed by BPE and customizing the compositional gradient, as shown in Fig. 4b. The mapping images show possible controlled isolation of the Mn zone by concentration. In the case of the Cu zone, keeping concentration constant leads to a repeatable zone formed at different runs. The increase in the concentration of Ni(NO_3_)_2_ (from 0.2 to 0.4 M) shows an increase in the deposition and relative expansion of the Ni zone. Similar behavior was observed upon increasing the concentration of Mn(NO_3_)_2_ from 0.2 to 0.4 M. The established method was also tested for isolating Cu, Co and Mn as a new metallic carpet on nickel foam. As shown in Fig. 5, the satisfactory results are confirmed by optical image, the respective elemental mapping analysis, and the EDX spectra (see Fig. S7), which approve the possibility of the rotational BPE for the further combination of metallic carpets aiming for different propositions.

The use of metal nitrate salts at neutral pH allows base generation and precipitation of metal oxides^41^. It seems that in the center of BPE, the Cu(II) ions reduce to Cu(I) and, subsequently, react with water to form a red-colored Cu_2_O^42^. Since the oxidation condition of Cu_2_O to CuO at the anodic half cycle is not provided around the central zone, the color does not change to black CuO. It is well known that Cu_2_O is not stable against corrosion at positive potentials in neutral pHs^43^. This is the main reason for the anodic dissolution of the Cu layer at the middle and border zones. Therefore, at high positive potentials of the anodic half-cycles, the electroplated Cu is restricted to the central zones, especially in higher DC potentials, as shown in Fig. 4a. Since the pH of electrolyte is not acidic, the anodic deposition of MnO_2_ does not occur, and it was assumed to be cathodically deposited as MnO^44,45^, as the static bipolar electroplating shows no observable Mn deposition during the anodic cylce.

To further confirm the in situ production of hydroxide anions, we conducted a blank BP experiment in the cell containing a solution of 1.2 M of KNO_3_ + 20 µl of phenolphthalein (PhP) indicator (the value of 1.2 M is equal to the total nitrate concentration resulting from three metal salts of Cu(NO_3_)_2_ = 0.02 M, Ni(NO_3_)_2_ = 0.2 and Mn(NO_3_)_2_ = 0.4 M). The colorless potassium nitrate-PhP solution was chosen so that we could easily observe the color change. A DC potential of 8 V was applied to the floated NF through the stainless steel driving electrodes. Upon turning on the power supply, the color of the local solution of the anodic poles of the driving and bipolar electrodes immediately changed to pink color, indicating the generation of the hydroxide ions via electroreduction of nitrate ions (Fig. S6). A similar experiment in the solution of colored transition metal salts, but due to the deep color of the environment, the resulting red color is not clear. On this basis, and based on the EDX spectrums (Figs. S4 and S5) that confirmed the presence of oxygen, we propose a well-known overall electrochemical reaction pathway as below^46^:

$${NO}_{3}^{-}+ {H}_{2}O+2e\underset{Cathodic\, reduction}{\longrightarrow }{NO}_{2}^{-}+2OH$$

$${M}^{n+}+nOH^{-} \to M\left(OH\right)n$$

$${M}^{n+}+nOH^{-}+\mathrm{O}_{2}\to MOx+H_{2}O$$

**Figure 6:**
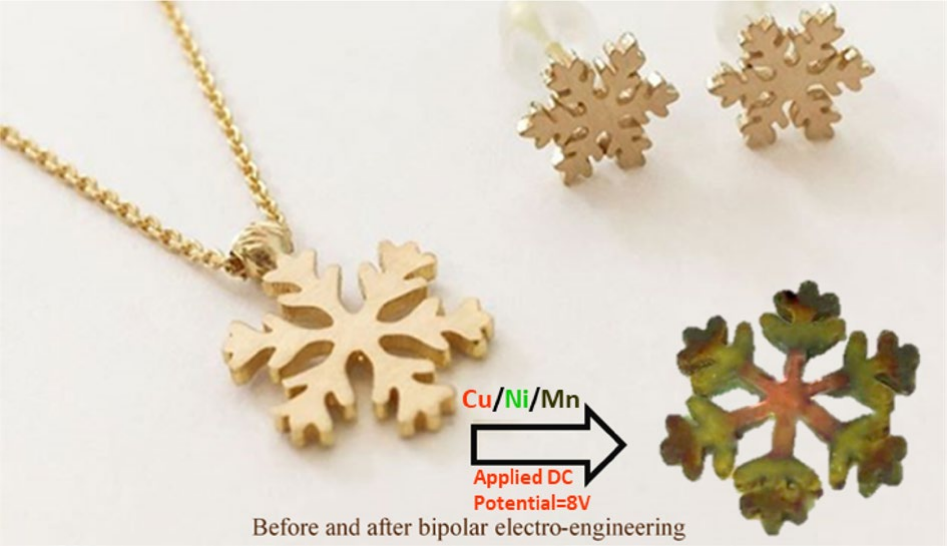
One-pot carpet-like bipolar electro-engineering used in future jewelry.

From a physical point of view, it seems that other equivalent physical occurrences can be effective in emerging these ring patterns. Upon the rotation of the BPE, as a conductor current flows through it, a magnetic field creates perpendicular to the current. This event causes a potential difference transverse to current flow in the electrical conductor (BPE), known as *Hall voltage*. The superposition of this potential on the already cell voltage can induce some nonlinearity in the potential of BPE. Hall voltage as a key factor can play a latent role in controlling the ring pattern and distributing charge species. In parallel, in the presence of a magnetic field, the charged species experience a force called the Lorentz force^47,48^. Without this magnetic field, the charges follow an approximate behavior in a straight path. While, upon applying a perpendicular magnetic field, their paths between collisions are curved and, thus, the moving charges accumulate on one face of the material. These fundamental challenges can become a subject for future studies of the effects involved in the formation of these concentric gradients. Further application of this procedure was tested for future jewelry, as shown in Fig. 6.

We perused the effect of higher rotating rates of 200, 300, 400, 600, 800, 1000, and 1200 rpm to gain additional information on the rotational electroplating of Cu, Ni, and Mn. The as-prepared carpets are shown in Fig. S8, where in all rotation rates, the concentric gradients were obtained, however, with the increase in rotation speed, some changes are obvious. The images show a gradual narrowing in Mn-margin upon increasing rotation rate, while the central copper zone does not change significantly. Along with the retreating of manganese, nickel expands to the edges of BPE. It is hypothesized that in parallel with the increase of rotation rates and switching frequency of the BPE poles, Mn deposition reaction with a relatively slower kinetic lagged behind the reduction reaction of Ni and Cu.

## Conclusions

This work discussed the preliminary studies performed on a new one-pot electro-engineering approach for the formation of two typical carpet-like ternary composites of Cu–Ni–Mn and Cu–Co–Mn at NFBPE. The effect of different physical parameters like DC potential, concentration polarization, kinetic polarizations, and rotation speed has been investigated to strongly support the observed changes in the pattern of metallic carpets. The results dedicated some exclusive features including, the one-pot formation of the wireless isolated concentric metallic composites, the simultaneous controlling of all zones of the compositional gradient, the ability to fabricate symmetrical composites on mobile electrodes in the electrolyte, and the creation of the compositional gradient over the surface of an electrode for future applications in artistic jewelry, nanomotor construction and so on. An ambitious goal of this idea is its future use as a cornerstone in materials engineering for multiple applications such as the design of new nanomotors, jewelry, electrochemical mechanistic studies, etc.

## Data availibility

The datasets used and/or analysed during the current study available from the corresponding author on reasonable request.

## Supplementary Information

Supplementary Information.
