# Supplementary material for: A one-pot rotational DC-bipolar approach for fabricating artistic metallic carpets
Source: Sci Rep. 2022 Oct 3;12:16537. doi: 10.1038/s41598-022-20929-7 (PMC9530174; doi:10.1038/s41598-022-20929-7)
Supplement: Supplementary file 1 — Supplementary Information. [file 41598_2022_20929_MOESM1_ESM.pdf]

Supplementary information for:

A one-pot rotational DC-bipolar approach for fabricating artistic metallic carpets

Fereshte Gholami[1], Mojtaba Shamsipur[1,*] and Afshin Pashabadi[1,*]

[1]Department of Chemistry, Razi University, Kermanshah, Iran.

Experimental

Materials and instruments

All chemicals were analytical grade and used without further purification. Nickel nitrate hexahydrate ($Ni(NO_3)_2$. $6H_2O$, 99%), manganese(II) nitrate tetrahydrate ($Mn(NO_3)_2$. $4H_2O$, 99%), copper(II) nitrate trihydrate ($Cu(NO_3)_2$. $3H_2O$, 99%), cobalt nitrate hexahydrate (Co $(NO_3)_2$. $6H_2O$, 99%), potassium nitrate (KNO_3), phenolphthalein, hydrochloric acid (HCl, 37%), ethanol (C_2H_5OH, 99%), acetone (C_3H_6O) were obtained from Sigma-Aldrich and Merck Co. All solutions were prepared with deionized water. The nickel foam (NF) and gold necklace (18 karats) were purchased from commercial sources. EDX spectrum and corresponding mapping analyses were obtained with a TESCAN MIRA3 HV apparatus operated at 20.0 kV from the Czech Republic. Optical images were obtained by a conventional cellphone camera (Samsung j7pro).

Overall Procedure for rotational bipolar electroplating on NF

The NF (10×12 mm) was carefully cleaned in 3 M HCl solution in an ultrasound bath for 15 min in order to remove the surface oxide layer. Then, it was immersed in acetone to remove organic materials from the surface. Finally, it was washed thoroughly with acetone, deionized water, and ethanol for 10 minutes respectively, and then was used as the BPE. It was located between two stainless steel driving electrodes (2.3 × 2 cm) in a square BP-cell (2.5 × 2.5 cm) containing a particular solution of varying amounts of metal ions. For rotation, the BE was attached to an RDE rotator (Metrohm) and rotated at 100 rpm. A constant DC potential was implemented from 4 to 12 V using a power supply (MASTECH DC power supply HY3005F-3) to the driving electrodes for a specific time (mostly 5 minutes) to obtain a metallic carpet.

Fabrication of typical Cu-Ni-Mn and Cu-Co-Mn carpet at NFBPE

A two-dimensional carpet-resemble compositional gradient of Cu-Ni-Mn was prepared by immersing the NF and the driving electrodes in an aqueous solution containing 0.4 M of $Mn(NO_3)_2$. $4H_2O$, 0.2 M of $Ni(NO_3)_2$. $6H_2O$ and 0.02 M $Cu(NO_3)_2$. $3H_2O$. The rotational DC-bipolar electrochemistry was done in several potentials (4-12 V) and rotational speed of 100 rpm. The optimum condition was obtained based on the visual inspection of fabricated metallic carpets. The carpet of Cu-Co-Mn was also prepared in an aqueous solution containing 0.4 M of $Mn(NO_3)_2$. $4H_2O$, 0.2 M of Co $(NO_3)_2$. $6H_2O$ and 0.02 M $Cu(NO_3)_2$. $3H_2O$ by employing the rotational DC-bipolar electrochemistry according to the discussed procedure.

Testing the rotational of bipolar electroplating in jewellery

An 18 karats gold necklace was used as the BPE. It was placed between two driving electrodes and attached to the motor controler tip, and rotated at 100 rpm. After 2 min, the attachment place was everted to make possible electroplating on the attachment place of BPE). It was immersed in an aqueous solution containing 0.4 M of $Mn(NO_3)_2$. $4H_2O$, 0.2 M of $Ni(NO_3)_2$. $6H_2O$ and 0.02 M $Cu(NO_3)_2$. $3H_2O$. A potential of 8 V was applied to the driving electrodes as the optimal potential for 5 mi for fabricating a carpet-like ternary couple of Cu-Ni-Mn on the gold BPE.

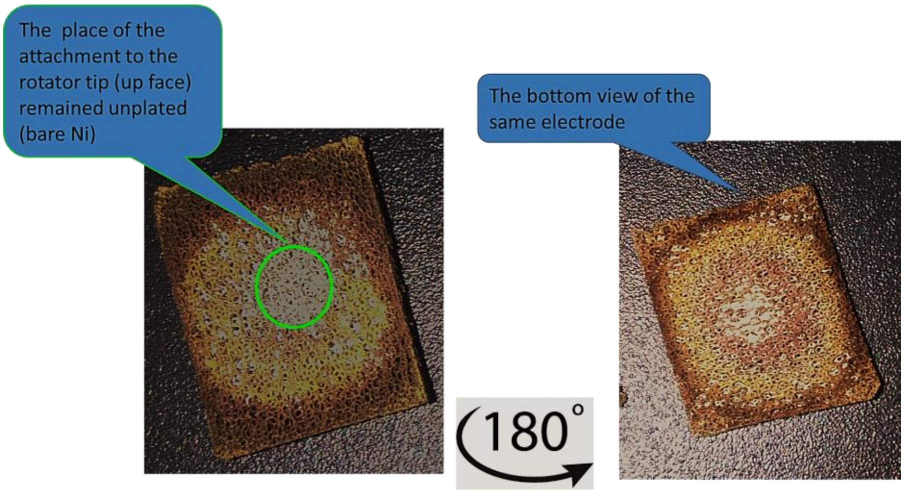

Figure S1. The optical image taken from the bottom and up faces of the BPE was subjected to rotational bipolar electroplating. The left image shows the place of the attachment to the rotator tip that remained partly colourless(Ni bare). The optical image shows an identical pattern formed on the back and front of the nickel foam as BPE.

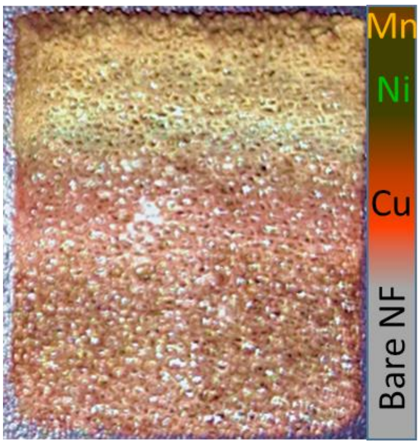

Figure S2. Static bipolar electroplating at 8 V for Cu, Ni and Mn. The image shows no anodic deposition at bottom part marked as bare NF.

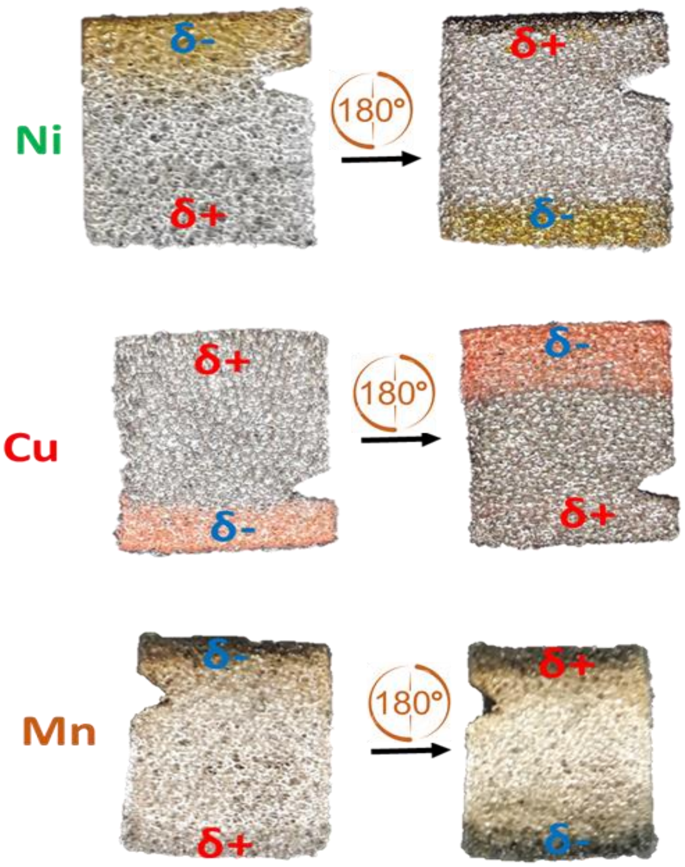

Figure S3. The study of anodic dissolution of the electroplated metallic oxides through static bipolar electroplating on a marked nickel foam. After separate deposition of either of Ni, Cu and Mn in solution containing each metal salt (for 2 minute). We rotated the poles 180° and further conducted wireless electroplating to investigate possible dissolution. Anodic dissolution occurred completely for Cu, partly for Ni and sparingly for Mn.

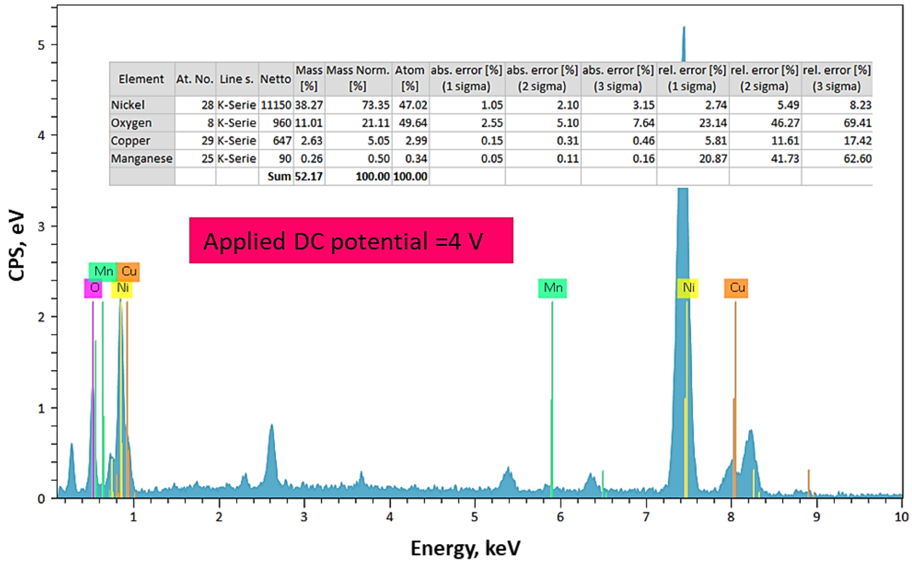

Element	At. No.	Line s.	Netto	Mass [%]	Mass Norm. [%]	Atom [%]	abs. error [%] (1 sigma)	abs. error [%] (2 sigma)	abs. error [%] (3 sigma)	rel. error [%] (1 sigma)	rel. error [%] (2 sigma)	rel. error [%] (3 sigma)
Nickel	28	K-Serie	11150	38.27	73.35	47.02	1.05	2.10	3.15	2.74	5.49	8.23
Oxygen	8	K-Serie	960	11.01	21.11	49.64	2.55	5.10	7.64	23.14	46.27	69.41
Copper	29	K-Serie	647	2.63	5.05	2.99	0.15	0.31	0.46	5.81	11.61	17.42
Manganese	25	K-Serie	90	0.26	0.50	0.34	0.05	0.11	0.16	20.87	41.73	62.60
			Sum	52.17	100.00	100.00						

Figure S4. EDX spectrum for Cu, Ni and Mn metallic carpet fabricated at applied DC potential of 4 V.

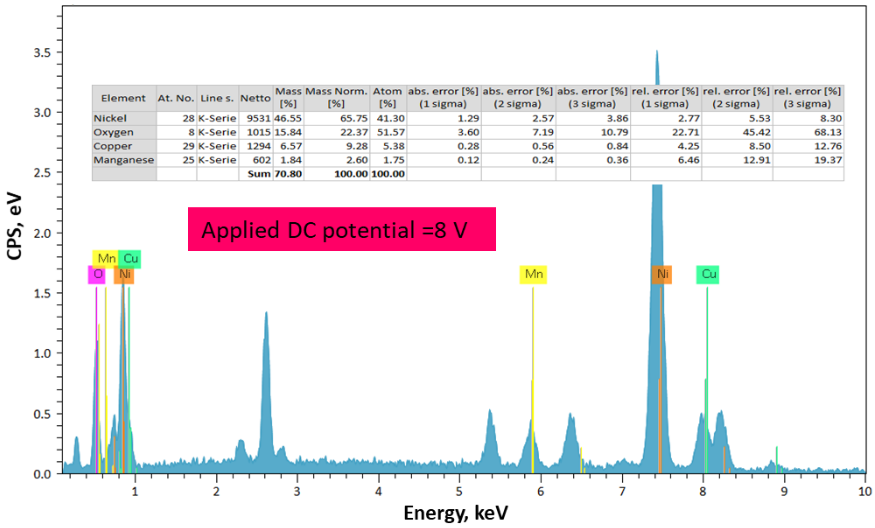

Element	At. No.	Line s.	Netto	Mass [%]	Mass Norm. [%]	Atom [%]	abs. error [%] (1 sigma)	abs. error [%] (2 sigma)	abs. error [%] (3 sigma)	rel. error [%] (1 sigma)	rel. error [%] (2 sigma)	rel. error [%] (3 sigma)
Nickel	28	K-Serie	9531	46.55	65.75	41.30	1.29	2.57	3.86	2.77	5.53	8.30
Oxygen	8	K-Serie	1015	15.84	22.37	51.57	3.60	7.19	10.79	22.71	45.42	68.13
Copper	29	K-Serie	1294	6.57	9.28	5.38	0.28	0.56	0.84	4.25	8.50	12.76
Manganese	25	K-Serie	602	1.84	2.60	1.75	0.12	0.24	0.36	6.46	12.91	19.37
			**Sum**	**70.80**	**100.00**	**100.00**						

Figure S5. EDX spectrum for Cu, Ni and Mn metallic carpet fabricated at applied DC potential of 8 V.

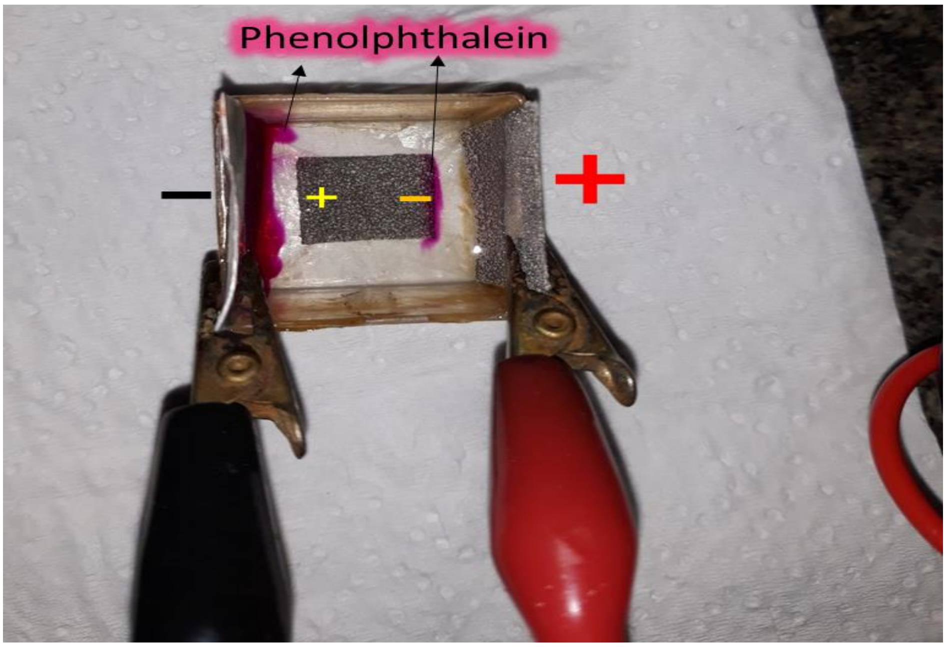

Figure S6. The aqueous solution containing KNO_3 with the total concentration equal to the nitrates of the mixed nitrate salt to simulate alkalinity of the cathode of the bipolar electrode immediately after turning down of the DC power supply. The pink color of the Phenolphthalein indicates possible formation of the oxy(hydroxide) of the Cu, Mn and Nickel at BPE.

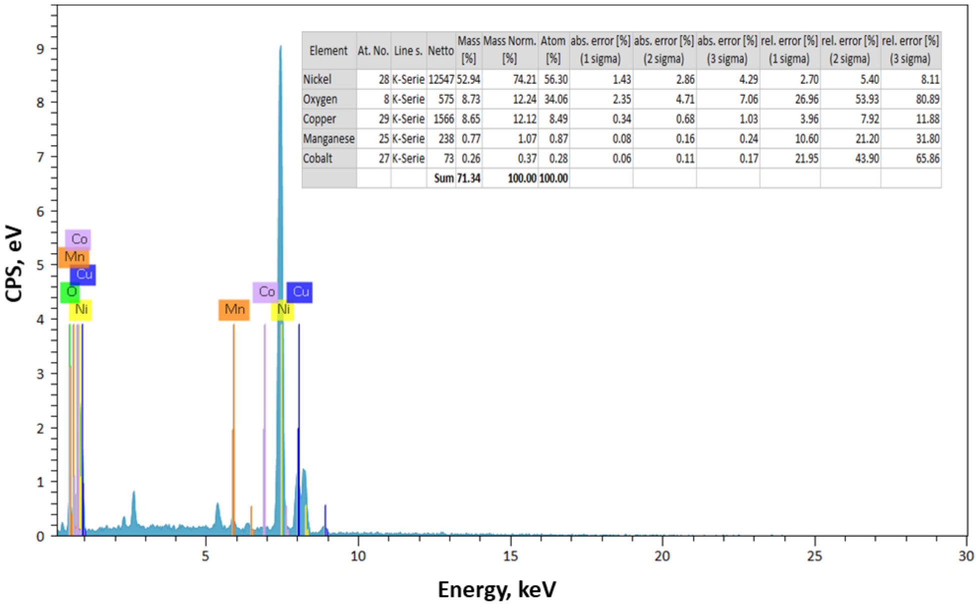

Element	At. No.	Line s.	Netto	Mass [%]	Mass Norm. [%]	Atom [%]	abs. error [%] (1 sigma)	abs. error [%] (2 sigma)	abs. error [%] (3 sigma)	rel. error [%] (1 sigma)	rel. error [%] (2 sigma)	rel. error [%] (3 sigma)
Nickel	28	K-Serie	12547	52.94	74.21	56.30	1.43	2.86	4.29	2.70	5.40	8.11
Oxygen	8	K-Serie	575	8.73	12.24	34.06	2.35	4.71	7.06	26.96	53.93	80.89
Copper	29	K-Serie	1566	8.65	12.12	8.49	0.34	0.68	1.03	3.96	7.92	11.88
Manganese	25	K-Serie	238	0.77	1.07	0.87	0.08	0.16	0.24	10.60	21.20	31.80
Cobalt	27	K-Serie	73	0.26	0.37	0.28	0.06	0.11	0.17	21.95	43.90	65.86
			Sum	**71.34**	**100.00**	**100.00**						

Figure S7. EDX spectrum for Cu, Co and Mn metallic carpet fabricated at applied DC potential of 8 V.

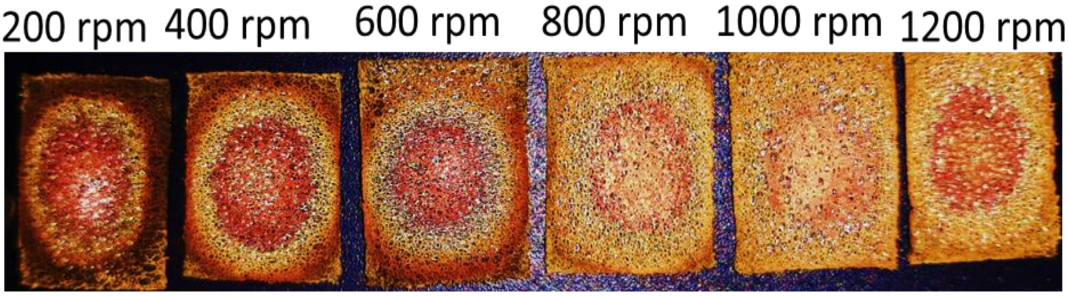

Figure S8. The effect of rotation speed of the motor controller on the fabricated pattern of Cu, Ni and Mn at DC potential of 8 V, for electroplating time of 5 minute, $Cu(NO_3)_2$=0.02 M, $Ni(NO_3)_2$ =0.2 and $Mn(NO_3)_2$=0.4 M.
